# Identification of meaningful individual-level change thresholds for worsening on the patient-reported outcomes version of the common terminology criteria for adverse events (PRO-CTCAE®)

**DOI:** 10.1007/s11136-024-03819-5

**Published:** 2024-11-06

**Authors:** Minji K. Lee, Sandra A. Mitchell, Ethan Basch, Gina L. Mazza, Blake T. Langlais, Gita Thanarajasingam, Brenda F. Ginos, Lauren Rogak, Eric A. Meek, Jennifer Jansen, Allison M. Deal, Philip Carr, Victoria S. Blinder, Mattias Jonsson, Gita N. Mody, Tito R. Mendoza, Antonia V. Bennett, Deborah Schrag, Amylou C. Dueck

**Affiliations:** 1 Alliance Foundation Trials Statistics and Data Center, Mayo Clinic, 200 1st Ave SW, Rochester, MN 55902 USA; 2 National Cancer Institute, Bethesda, MD USA; 3 Lineberger Comprehensive Cancer Center, University of North Carolina at Chapel Hill, Chapel Hill, NC USA; 4 Alliance Foundation Trials Statistics and Data Center, Mayo Clinic, Scottsdale, AZ USA; 5 Department of Quantitative Health Sciences, Mayo Clinic, Scottsdale, AZ USA; 6 Division of Hematology, Mayo Clinic, Rochester, MN USA; 7 Memorial Sloan Kettering Cancer Center, New York, NY USA

**Keywords:** Individual-level meaningful change threshold, PRO-CTCAE, Meaningful worsening

## Abstract

**Background:**

We derived meaningful individual-level change thresholds for worsening in selected patient-reported outcomes version of the common terminology criteria for adverse events (PRO-CTCAE®) items and their composite scores.

**Methods:**

We used two data sources, the PRO-TECT trial (Alliance AFT-39) that collected PRO-CTCAE data from adults with advanced cancer at 26 United States (U.S.) community oncology practices and the PRO-CTCAE validation study that collected PRO-CTCAE data from adults undergoing chemotherapy or radiation therapy at nine U.S. cancer centers or community oncology practices. Both studies administered selected PRO-CTCAE items and EORTC QLQ-C30 scales. Conceptually, relevant QLQ-C30 domains were used as anchors to estimate meaningful change thresholds for deterioration in corresponding PRO-CTCAE items and their composite scores. Items or composites with ǀ*ρ*ǀ ≥ 0.30 correlation with QLQ-C30 scales were included. Changes in PRO-CTCAE scores and composites were estimated for patients who met or exceeded a 10-point deterioration on the corresponding QLQ-C30 scale. Change scores were computed between baseline and the 3-month timepoint in PRO-TECT, and in the PRO-CTCAE validation study between baseline and a single follow-up visit that occurred between 1 and 7 weeks later. For each PRO-CTCAE item, change scores could range from − 4 to 4; for a composite, change scores could range from − 3 to 3.

**Results:**

Change scores in QLQ-C30 and PRO-CTCAE were available in 406 and 792 patients in PRO-TECT and the validation study, respectively. Across QLQ-C30 scales, the proportion of patients with a 10-point or greater worsening on QLQ-C30 ranged from 15 to 30% in the PRO-TECT data and 13% to 34% in the validation data. Across PRO-CTCAE items, anchor-based meaningful change estimates for deterioration ranged from 0.05 to 0.30 (mean 0.19) in the PRO-TECT data and from 0.19 to 0.53 (mean 0.36) in the validation data. For composites, they ranged from 0.06 to 0.27 (mean 0.17) in the PRO-TECT data and 0.22 to 0.51 (mean 0.37) in the validation data.

**Conclusion:**

In both datasets, the minimal meaningful individual-level change threshold for worsening was one point for all items and composite scores.

**ClinicalTrials.gov:** NCT03249090 (AFT-39), NCT02158637 (MC1091)

**Supplementary Information:**

The online version contains supplementary material available at 10.1007/s11136-024-03819-5.

## Introduction

Short-term toxicities can arise at any time during cancer treatment, and long-term and late effects can persist or arise beyond active treatment. The purpose of the Patient-Reported Outcomes version of the Common Terminology Criteria for Adverse Events (PRO-CTCAE®) is to capture symptomatic adverse events by patient self-report in cancer clinical trials [1]. PRO-CTCAE is designed to complement clinician adverse event reporting using Common Terminology Criteria for Adverse Events (CTCAE).

A prior study demonstrated that each ordinal response choice for PRO-CTCAE distinguished respondents with meaningfully different symptom experiences [2]. Because each CTCAE grade can inform clinical actions, a prior study used any 1-point score change in PRO-CTCAE as a meaningful change [3]. However, empirical evidence is lacking to support the assumption that a score change of one point is meaningful. The design, analysis, and interpretation of studies using PRO-CTCAE can be enhanced by deriving meaningful change thresholds (MCTs) to identify important changes for individuals.

Change scores in the European Organization for Research and Treatment of Cancer Quality of Life Questionnaire-Core 30 (EORTC QLQ-C30), a 30-item instrument for assessing quality of life in cancer patients, have been frequently used as anchors to derive clinically important differences and meaningful change thresholds for other patient-reported outcome measures [4, 5]. An individual-focused approach requires an anchor at the individual level to determine which changes are important for individual patients [6]. Patients can have their own individual thresholds of a minimally important change (MIC) and the mean of these individual MIC thresholds can be conceptualized as the average within-person MIC [7, 8]. Using the EORTC QLQ-C30 as the anchor and the predictive modeling method, in this analysis we derived minimally important individual-level change thresholds for worsening in PRO-CTCAE.

## Methods

### Measures

PRO-CTCAE is an item library that includes 78 symptomatic adverse events (AEs), which are each assessed by one to three attribute questions (i.e., representing the frequency, severity, and/or interference; amount or presence of the AE), yielding a total of 124 individual items [1]. Each item is rated using a five-level verbal descriptor scale, resulting in integer scores ranging from 0 to 4. The EORTC QLQ-C30 is a 30-item questionnaire that generates a global health status/quality of life (QOL) scale score, five functioning scale scores, and eight symptom item/scale scores (excluding financial difficulties score) ranging between 0 and 100. The QLQ-C30 summary score is calculated from the mean of the combined 13 QLQ-C30 scale and item scores (excluding the global health status/QOL and the financial difficulties) [9–11]. All EORTC scales were coded such that higher scores indicate worse symptoms, functioning, and quality of life. The recall period for both PRO-CTCAE and the EORTC QLQ-C30 is the past week.

### Data sources

#### PRO-CTCAE validation data

The PRO-CTCAE validation data in this study refers to the data collected previously as part of a PRO-CTCAE validation study conducted in collaboration with the US National Cancer Institute (NCT02158637). Adult patients initiating or undergoing chemotherapy, radiation therapy, or both at nine United States (U.S.) cancer centers or community oncology practices were invited to participate in the study [12]. Eligible patients were approached in clinic waiting areas to participate, and their written informed consent was obtained. This study implemented a survey administration design as illustrated in eTable 1 of Dueck et al. [12]. Initially, until 80% of the sample was accrued, all participants received items related to the 21 most commonly occurring symptomatic AEs across cancer types [13, 14], along with specific items assigned to each cancer type. For instance, patients with head/neck or esophageal cancer answered questions about 21 common AEs and additional symptoms like blurry vision, cough, and difficulty swallowing. After reaching 80% of the accrual goal, the focus shifted from the 21 common AE terms to the remaining AE terms, ensuring comprehensive coverage by administering these items to all participants. Our investigation concentrated on the PRO-CTCAE items associated with the 21 commonly occurring symptomatic AEs due to their relatedness to the EORTC QLQ-C30 measures that were used as anchors. We used the PRO-CTCAE items and EORTC QLQ-C30 that were administered at baseline and at a follow-up visit, which took place between 1 and 7 weeks later.

#### PRO-TECT trial

The PRO-TECT (Patient Reported Outcomes to Enhance Cancer Treatment) trial (AFT-39) enrolled adult individuals with advanced cancer to respond to PRO measures during treatment at 52 U.S. community oncology practices (NCT03249090) [15]. Adults undergoing treatment with chemotherapy, targeted therapy, or immunotherapy for any form of advanced or metastatic cancer within these practices were invited and provided informed consent to join the study. We used the data from 26 sites that remotely administered PRO measures that patients completed weekly from home. The trial engaged diverse stakeholders, including patients with cancer, clinicians, health services researchers, a scientific advisory board, and committees from the Alliance for Clinical Trials in Oncology to select key symptoms and PRO measures, among which were the PRO-CTCAE items and EORTC QLQ-C30 scales. The PRO-CTCAE items assessed the frequency (F), severity (S), and/or interference (I) of the following 11 items weekly: pain (FSI), diarrhea (F), constipation (S), nausea (FS), vomiting (F), insomnia (S), and shortness of breath (SI). These seven AE terms, associated with the 11 items, are part of the 21 commonly occurring AEs, and all participants received all 11 items each week. The EORTC QLQ-C30 was administered at baseline and at 1, 3, 6, 9, and 12 months. For this analysis, we examined the baseline and 3-months follow-up data based on the number of available survey responses and the strength of the correlations with the PRO-CTCAE items. For PRO-CTCAE items, responses provided at the week 12 (3-month) assessment were analyzed.

### Statistical analyses

An anchor-based approach was used to derive the average individual-level MIC thresholds, involving mapping change from baseline in the target measure to other assessments of change (i.e., the anchors). We used the EORTC QLQ-C30 symptom or function scales as anchors to derive the MIC thresholds for select PRO-CTCAE items (i.e., the target measure). We included in our analysis those PRO-CTCAE items where the change scores on PRO-CTCAE and the conceptually relevant EORTC QLQ-C30 scale demonstrated a Spearman correlation of at least |0.30| [16, 17]. We defined a respondent a ‘decliner’ if they met or exceeded a 10-point worsening from baseline to 3 months on the relevant EORTC QLQ-C30 scale (i.e., the anchor). Prior research [18] has supported a score change of 10-points on the EORTC as a threshold for interpreting meaningful within-patient changes.

#### MIC predictive modeling of PRO-CTCAE items

We used the MIC predictive modeling method [8, 19] to estimate the predictive probability that a patient belongs to the worsened group (based on the relevant EORTC QLQ-C30 scale) given the observed PRO-CTCAE change score. This method uses logistic regression analysis with the individual-level change scores on PRO-CTCAE as the predictor variable and the group membership (worsened versus not worsened on the EORTC anchor item/scale) as the dependent variable. The logistic regression equation is:

$${\text{ln}}\left( {{\text{odds}}_{{{\text{post}}}} } \right) \, = C + B_{X} \times X,$$

where odds_post_ are the odds of belonging to the group that experienced a decline in symptoms or functioning (referred to as the worsened group) given the PRO-CTCAE change score; *C* represents the intercept; *B*_*X*_ the regression coefficient of the change score *X*.

The MIC for worsening is defined as the change score associated with a likelihood ratio (LR) of 1, where the probability of belonging to the worsened group after knowing the PRO-CTCAE change score (odds_post_) equals the probability of belonging to the worsened group before knowing the PRO-CTCAE change score (odds_pre_). The latter probability, odds_pre_ is based on the anchor and is determined independently of PRO-CTCAE. LR > 1 (LR < 1) indicates that probability of belonging to the worsened group given the PRO-CTCAE change score is greater (smaller) than probability of being in the worsened group based on the anchor. Because LR = odds_post_/odds_pre_, odds_post_ equals odds_pre_ when LR = 1. Substituting odds_post_ with odds_pre_ yields

$${\text{ln}}\left( {{\text{odds}}_{{{\text{pre}}}} } \right) \, = C + B_{X} \times X$$

$$X \, = \left( {{\text{ln}}\left( {{\text{odds}}_{{{\text{pre}}}} } \right) \, - C} \right)/B_{X}$$

*X* is the unadjusted estimate of MIC for worsening, which provides an unbiased estimate under the scenario that the proportion worsened equals the proportion not worsened on the anchor (i.e., *p* = 0.5), such that ln(odds_pre_) = 0. Because there is downward bias of the MIC when proportion worsened in the sample < 0.5, we adjusted the MIC using the following formula [7]:

$${\text{MIC}}_{{{\text{adjusted}}}} = {\text{ MIC}}_{{{\text{unadjusted}}}} {-} \, \left( {0.0{9 } + \, 0.{1}0{3} \times Cor} \right) \, \times {\text{ SD}}_{{{\text{change}}}} \times {\text{ ln}}\left( {{\text{odds}}_{{{\text{pre}}}} } \right),$$

where *Cor* is the point-biserial correlation between the PRO-CTCAE change score and the dichotomized anchor (i.e., worsened vs not-worsened); SD_change_ is the standard deviation of the PRO-CTCAE change score; and ln(odds_pre_) is the natural logarithm of proportion worsened on the anchor divided by one minus proportion worsened. We report the MIC_adjusted_ for all MIC analyses, and bootstrapping (1000 samples) was used to calculate confidence intervals.

#### MIC predictive modeling for PRO-CTCAE composite scores

A single composite numerical score ranging from 0 to 3 for each PRO-CTCAE symptomatic adverse event was computed based on the published algorithm [20]. The MICs on the composite scores that had *ρ* values greater than 0.30 with related QLQ-C30 scales were derived using the predictive modeling.

#### Average changes in PRO-CTCAE items and composites for decliners

We calculated the mean PRO-CTCAE change scores for the decliner group (i.e., those who met or exceeded a 10-point decline on the relevant EORTC QLQ-C30 scale). This mean method is a simple and commonly used approach to estimate the MIC values at the group level. Although the value may not provide a threshold for minimal deterioration because it is the mean of the entire group of decliners, we provide these results for comparison with the MIC_adjusted_ values.

## Results

### Sample characteristics

Table 1 shows the participant baseline characteristics for the two datasets. 406 participants out of 590 in the PRO-TECT dataset and 792 participants out of 852 in the PRO-CTCAE validation dataset contributed to at least one analysis reported in this paper.

**Table 1:** Participant baseline characteristics

	PRO-TECT data (N = 406)	Validation data (N = 792)
Age at enrollment (years)		
Mean (SD)	61.7 (11.55)	58.0 (12.79)
Median	63.0	59.0
Range	29.0, 88.0	19.0, 90.0
Gender, n (%)		
Female	255 (62.8%)	446 (56.3%)
Male	151 (37.2%)	346 (43.7%)
Race, n (%)		
White	325 (80.0%)	571 (72.1%)
Black or African American	69 (17.0%)	170 (21.5%)
Native Hawaiian	0 (0%)	3 (0.4%)
Asian	2 (0.5%)	38 (4.8%)
Native American	6 (1.5%)	2 (0.3%)
Multiple races reported	1 (0.2%)	1 (0.1%)
Unknown/not reported	3 (0.7%)	7 (0.9%)
Ethnicity, n (%)		
Hispanic/Latino	8 (2.0%)	48 (6.1%)
Non-Hispanic	398 (98.0%)	703 (88.8%)
Unknown/not reported	0 (0%)	41 (5.2%)
Education level, n (%)		
Less than high school	28 (6.9%)	55 (6.9%)
High school or GED	117 (28.8%)	202 (25.5%)
Some college	162 (39.9%)	177 (22.3%)
College graduate or more	99 (24.4%)	352 (44.4%)
Missing	0 (0%)	6 (0.8%)
Disease, n (%)		
Breast	71 (17.5%)	225 (28.4%)
Head/neck	0 (0%)	129 (16.3%)
Lung	82 (20.2%)	158 (19.9%)
Gastrointestinal	108 (26.6%)	79 (10.0%)
Genitourinary/gynecology	96 (23.6%)	144 (18.2%)
Hematologic	24 (5.9%)	36 (4.5%)
Other/unknown	25 (6.2%)	21 (2.7%)
ECOG PS (Visit 1), n (%)		
ECOG 0–1	374 (92.1%)	666 (84.1%)
ECOG 2–4	32 (7.9%)	126 (15.9%)
Line of systemic cancer treatment at baseline, n (%)		
1st	132 (32.5%)	
2nd	132 (32.5%)	
3rd	72 (17.7%)	
4th or later	70 (17.2%)	
Intravenous delivery of cancer treatment at baseline, n (%)	359 (88.4%)	
Oral delivery of cancer treatment at baseline, n (%)	71 (17.5%)	
Treatment at Visit 1, n (%)^a^		
Radiation therapy in past 2 weeks		380 (48.0%)
Surgery in past 2 weeks		30 (3.8%)
Chemotherapy in past 2 weeks		439 (55.4%)

406 patients in the PRO-TECT dataset and 792 patients in the validation dataset contributed to at least one analysis. To be included in this study, patients needed to have at least one pair of PRO-CTCAE item change scores and the corresponding change scores on EORTC QLQ-C30 scales

*GED* general educational development

^a^The percentages do not add up to 100% since some participants had received more than one treatment modality in the past 2 weeks

#### PRO-TECT trial

The sample median age was 63 years, and about 63% were female, 80% white, 2% Hispanic/Latino, and 76% had some college or below. About 27% had gastrointestinal cancer, 24% genitourinary or gynecologic cancer, 20% lung, and 18% breast cancer. A majority (92%) had an Eastern Cooperative Oncology Group (ECOG) performance status 0–1.

#### PRO-CTCAE validation study

The sample median age was 59 years, and about 56% were female, 72% white, 6% Hispanic/Latino, and 55% had some college or below. About 28% had breast cancer, 20% lung, 18% genitourinary or gynecologic cancer, and 16% head/neck. A majority (84%) had an ECOG performance status 0–1.

### Selecting PRO-CTCAE items for MIC analyses

Supplementary Tables 1 and 2 present the correlations between the change scores from baseline to 3 months in PRO-CTCAE items and change scores in conceptually related EORTC QLQ-C30 scales. For the PRO-TECT data, all correlations between PRO-CTCAE change scores and EORTC QLQ-C30 change scores exceeded *ρ* of 0.30 (Supplementary Table 1). Based on these correlations, the MICs on the following items were investigated in the PRO-TECT data: Pain (FSI), nausea (FS), vomiting (F), constipation (S), diarrhea (F), shortness of breath (SI), and insomnia (S). For the PRO-CTCAE validation data, the following items were included for MIC analysis based on *ρ* values greater than 0.30 (Supplementary Table 2): Anxious (FSI), constipation (S), decreased appetite (SI), fatigue (SI), insomnia (SI), diarrhea (F), nausea (FS), pain (FSI), concentration (SI), taste changes (S), sad (F), shortness of breath (S), and vomiting (FS).

### Worsening by at least 10 points on the EORTC anchor scales

**Fig. 1:**
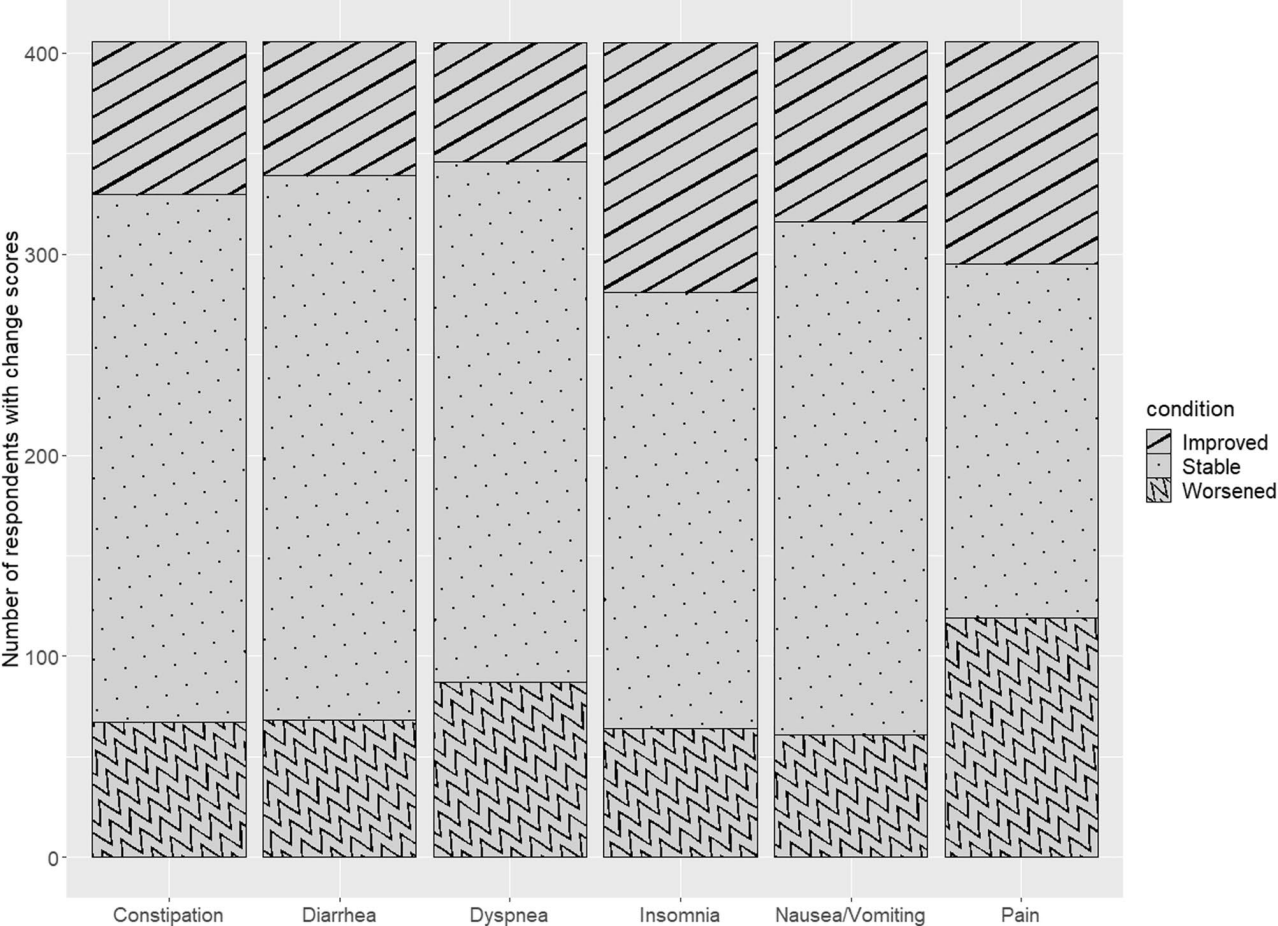
PRO-TECT data: Number of respondents who improved, stayed stable, or worsened (based on 10-point change) at 3-month follow-up in EORTC QLQ-C30 scales that were used as anchors. The analysis sample from the PRO-TECT data consisted of 406 patients who contributed to at least one analysis. Based on 10-point worsening in QLQ-C30 scores, 16.5% reported worsening constipation, 16.7% worsening diarrhea, 21.5% worsening dyspnea, 15.8% worsening insomnia, 15.0% worsening nausea/vomiting, and 29.3% worsening pain

**Fig. 2:**
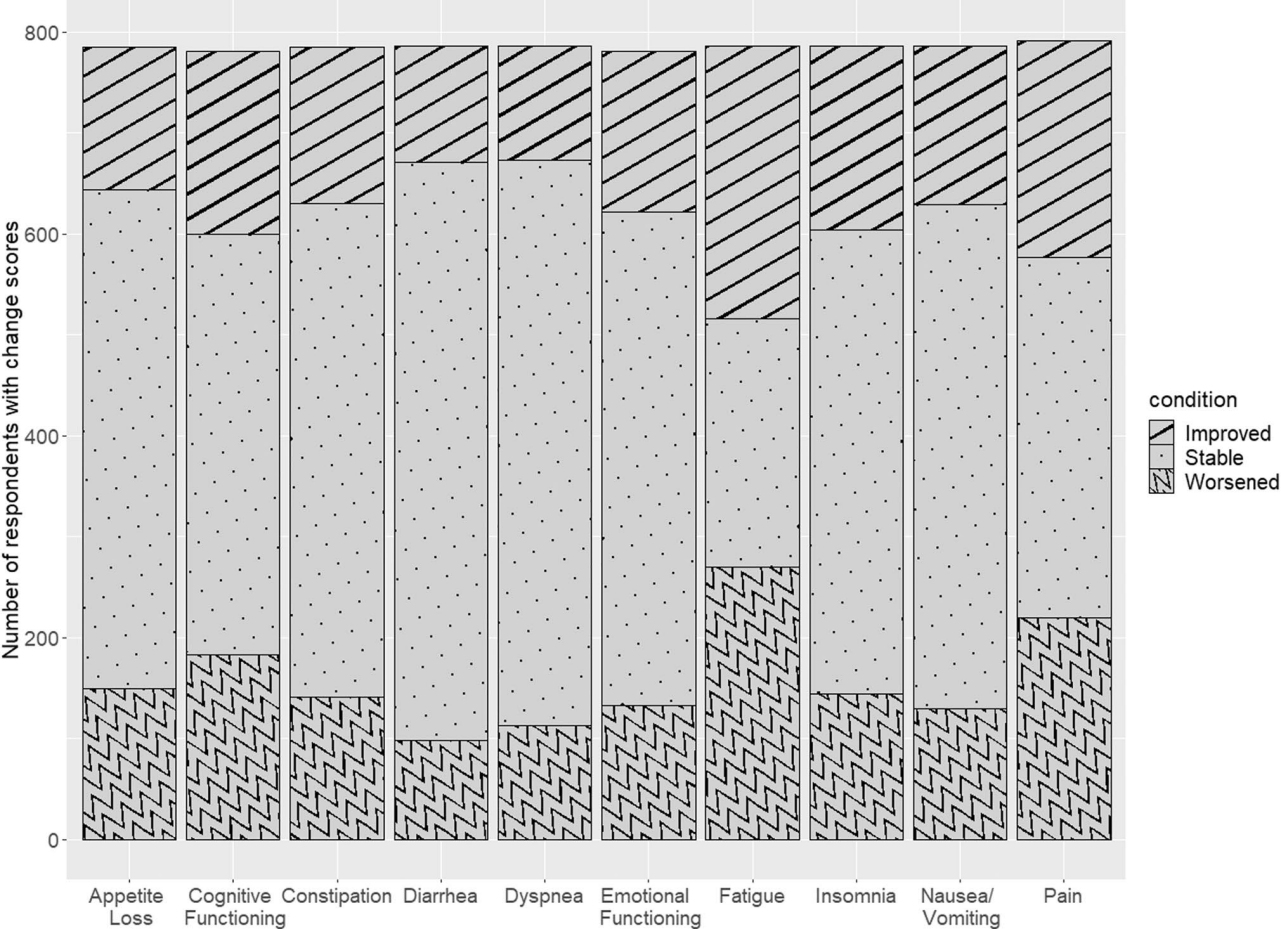
Validation data: number of respondents who improved, stayed stable, or worsened (based on 10-point change) in EORTC QLQ-C30 scales that were used as anchors. The validation data analysis sample consisted of 792 patients who contributed to at least one analysis. Using the anchors of EORTC QLQ-C30 scales, those who worsened by 10 points or more were categorized as decliners. There were 19.1% who reported worsening appetite loss, 23.4% declining cognitive function, 18.0% worsening constipation, 12.5% worsening diarrhea, 14.4% worsening dyspnea, 17.0% declining emotional function, 34.4% worsening fatigue, 18.4% worsening insomnia, 16.5% worsening nausea/vomiting, and 27.8% worsening pain

In the PRO-TECT data, using the 10-point EORTC score threshold for interpreting within-patient changes, 16.5% reported worsening constipation, 16.7% worsening diarrhea, 21.5% worsening dyspnea, 15.8% worsening insomnia, 15.0% worsening nausea/vomiting, and 29.3% worsening pain (Fig. 1). In the validation data, 19.1% reported worsening appetite loss, 23.4% declining cognitive function, 18.0% worsening constipation, 12.5% worsening diarrhea, 14.4% worsening dyspnea, 17.0% declining emotional function, 34.4% worsening fatigue, 18.4% worsening insomnia, 16.5% worsening nausea/vomiting, and 27.8% worsening pain (Fig. 2).

### MIC predictive modeling of PRO-CTCAE observed scores

As shown in Table 2, in the PRO-TECT dataset, PRO-CTCAE MIC estimates ranged from 0.05 to 0.30 with a mean of 0.19 (median 0.20), and the upper limits of their 95% confidence intervals ranged from 0.20 to 0.45 with a mean of 0.32 (median 0.32). In the validation data, the MIC estimates ranged from 0.19 to 0.53 with a mean of 0.36 (median 0.34), and the upper limits of their 95% confidence intervals ranged from 0.27 to 0.66 with a mean of 0.46 (median 0.46).

**Table 2:** MICs in PRO-CTCAE items

Anchor: EORTC QLQ-C30 scale	PRO-CTCAE item	MIC in the PRO-TECT data (95% confidence interval)	MIC in the validation data (95% confidence interval)
Emotional functioning	Anxious (F)		0.37 (0.25, 0.50)
	Anxious (S)		0.31 (0.20, 0.42)
	Anxious (I)		0.34 (0.22, 0.46)
	Sad (F)		0.30 (0.18, 0.42)
Constipation	Constipation (S)	0.10 (− 0.05, 0.25)	0.49 (0.39, 0.60)
Appetite loss	Decreased appetite (S)		0.53 (0.42, 0.63)
	Decreased appetite (I)		0.41 (0.29, 0.52)
	Taste changes (S)		0.43 (0.28, 0.56)
Fatigue	Fatigue (S)		0.24 (0.17, 0.31)
	Fatigue (I)		0.19 (0.11, 0.27)
Insomnia	Insomnia (S)	0.18 (0.03, 0.32)	0.42 (0.32, 0.52)
	Insomnia (I)		0.35 (0.24, 0.46)
Diarrhea	Diarrhea (F)	0.30 (0.15, 0.45)	0.53 (0.39, 0.66)
Nausea or vomiting	Nausea (F)	0.25 (0.06, 0.42)	0.51 (0.40, 0.61)
	Nausea (S)	0.20 (0.06, 0.35)	0.46 (0.37, 0.56)
	Vomiting (F)	0.20 (0.12, 0.30)	0.30 (0.21, 0.39)
	Vomiting (S)		0.29 (0.21, 0.39)
Pain	Pain (F)	0.05 (− 0.08, 0.20)	0.34 (0.25, 0.42)
	Pain (S)	0.09 (− 0.03, 0.22)	0.30 (0.21, 0.39)
	Pain (I)	0.11 (− 0.02, 0.23)	0.30 (0.20, 0.39)
Cognitive functioning	Concentration (S)		0.26 (0.16, 0.35)
	Concentration (I)		0.25 (0.15, 0.33)
Dyspnea	Shortness of breath (S)	0.30 (0.19, 0.40)	0.42 (0.33, 0.50)
	Shortness of breath (I)	0.29 (0.19, 0.39)	–

Using the anchors of EORTC QLQ-C30 scales, those who worsened by 10 points or more were categorized as decliners. MICs of the selected PRO-CTCAE items in the PRO-TECT data ranged from 0.05 to 0.30 with mean of 0.20 (median: 0.20). Those in the validation data ranged from 0.19 to 0.53 with mean of 0.36 (median: 0.34). The blanks in the table show the PRO-CTCAE items that we did not administer in the PRO-TECT trial

The “–” shows the item that had a low correlation (≤ 0.30) with the anchor

### PRO-CTCAE composite scores for MIC analyses

Using the PRO-TECT data, composite scores were calculated for five PRO-CTCAE symptomatic adverse events: constipation, diarrhea, dyspnea, nausea, and pain. For all five composites, the Spearman *ρ* between EORTC change scores and PRO-CTCAE composite change scores exceeded 0.30 (Table 3), ranging from 0.38 to 0.47. In the validation data, composite scores for 12 symptomatic adverse events met the criterion of *ρ* > 0.30 (Table 4).

**Table 3:**
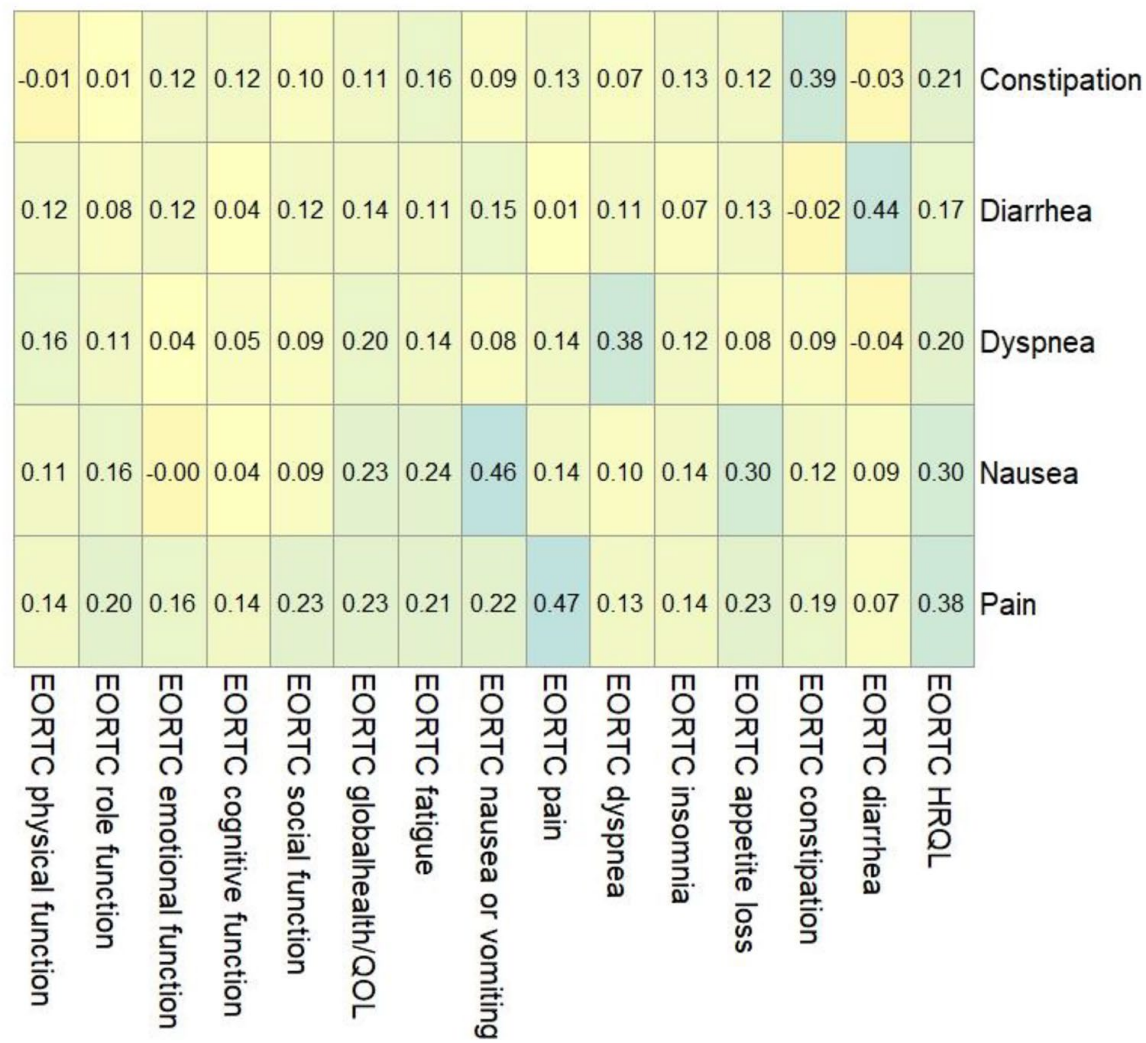
PRO-TECT data: correlations between the changes in PRO-CTCAE composite scores and changes in EORTC scales

All EORTC scales were coded such that higher scores indicate worse symptoms, functioning, and quality of life. Based on these correlations, the MICs in the following composite scores were investigated in the PRO-TECT data: constipation, diarrhea, dyspnea, nausea, and pain

**Table 4:**
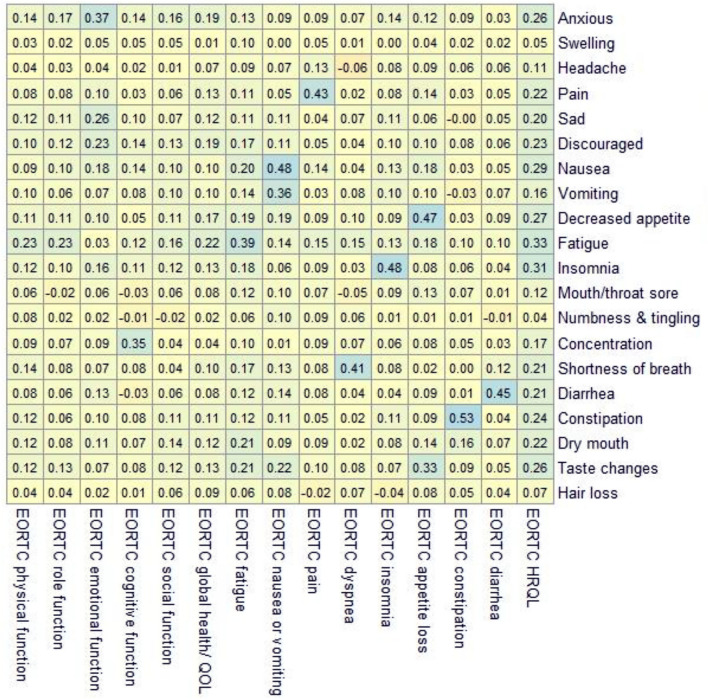
Validation data: correlations between the changes in PRO-CTCAE composite scores and changes in EORTC scales (composites for which at least 85% of the patients have change scores were included in the correlation analysis)

All EORTC scales were coded such that higher scores indicate worse symptoms, functioning, and quality of life. Based on these correlations, the MICs in the following composite scores were investigated in the validation data: anxious, pain, nausea, vomiting, decreased appetite, fatigue, insomnia, concentration, shortness of breath, diarrhea, constipation, and taste changes

### MIC predictive modeling of PRO-CTCAE composite scores

As shown in Table 5, in the PRO-TECT data, the MIC estimates ranged from 0.06 to 0.27 with a mean of 0.17 (median 0.21), and the upper limits of their 95% confidence intervals ranged from 0.18 to 0.36 with a mean of 0.28 (median 0.31). In the validation data, the MIC estimates ranged from 0.22 to 0.51 with a mean of 0.37 (median 0.38), and the upper limits of their 95% confidence intervals ranged from 0.30 to 0.63 with a mean of 0.48 (median 0.50).

**Table 5:** MICs in PRO-CTCAE composite scores

Anchor: EORTC QLQ-C30 scale	PRO-CTCAE composite score	MIC in the PRO-TECT data (95% confidence interval)	MIC in the validation data (95% confidence interval)
Emotional functioning	Anxious		0.30 (0.16, 0.41)
Constipation	Constipation	0.08 (− 0.04, 0.22)	0.50 (0.36, 0.63)
Appetite loss	Decreased appetite		0.49 (0.37, 0.61)
	Taste changes		0.37 (0.20, 0.53)
Fatigue	Fatigue		0.22 (0.13, 0.30)
Insomnia	Insomnia		0.51 (0.38, 0.63)
Diarrhea	Diarrhea	0.21 (0.11, 0.31)	0.39 (0.28, 0.49)
Nausea or vomiting	Nausea	0.21 (0.08, 0.35)	0.47 (0.36, 0.58)
	Vomiting		0.25 (0.15, 0.34)
Pain	Pain	0.06 (− 0.06, 0.18)	0.32 (0.23, 0.41)
Cognitive functioning	Concentration		0.26 (0.16, 0.36)
Dyspnea	Shortness of breath	0.27 (0.18, 0.36)	0.40 (0.28, 0.50)

Using the anchors of EORTC QLQ-C30 scales, those who worsened by 10 points or more were categorized as decliners. MICs of the selected PRO-CTCAE composite scores in the PRO-TECT data ranged from 0.06 to 0.27 with mean of 0.17 (median: 0.20). Those in the validation data ranged from 0.22 to 0.51 with mean of 0.37 (median: 0.38). The blanks in the table show the composites in the PRO-TECT data, for which we did not have the complete set of items in the dataset

### Classification accuracy in EORTC QLQ-C30 with PRO-CTCAE for identifying worsening

The classification accuracy, sum of true positives and true negatives, was computed by calculating the percentages of agreement on worsening and not worsening by change in EORTC QLQ-C30 (> 10 vs. ≤ 10) and change in PRO-CTCAE (> 0 vs. ≤ 0). Across the PRO-CTCAE items, classification accuracy in the PRO-TECT data ranged from 61 to 86% with a mean of 78% (median 79%) for the PRO-CTCAE items. Using the PRO-CTCAE validation dataset, accuracy ranged from 69 to 87% with a mean of 80% (median 79%). For the composite scores, classification accuracy in the PRO-TECT data ranged from 76 to 85% with a mean of 81% (median 82%); in the validation dataset, accuracy ranged from 70 to 87% with a mean of 82% (median 82%).

#### Average changes in PRO-CTCAE items and composite scores for decliners

In the PRO-TECT data, the average change scores for decliners ranged from 0.22 to 0.53 with a mean of 0.39 (median 0.36); in the validation data, average change scores ranged from 0.32 to 0.96 with a mean of 0.61 (median 0.57) (Supplementary Table 3). The average changes in PRO-CTCAE composite scores ranged from 0.19 to 0.47 with a mean of 0.34 (median 0.35) in the PRO-TECT dataset; those in the validation data ranged from 0.36 to 0.85 with a mean of 0.56 (median 0.55) (Supplementary Table 4). Average change scores for the PRO-CTCAE observed scores and composite scores were below 1 for symptomatic adverse events examined in this study.

## Discussion

In this study, we found that a one-point change reflects meaningful individual-level worsening for all PRO-CTCAE items investigated. Across different symptomatic adverse events, we demonstrated, using two datasets, that PRO-CTCAE MIC estimates for worsening were 0.53 or below, with the upper limits of the 95% confidence intervals at 0.66 or below. Given the ordinal nature of the PRO-CTCAE items, for the subset of PRO-CTCAE items examined in this study, a one-point increase represents a minimally important individual-level change that reflects meaningful worsening. Similarly, across symptomatic adverse events and datasets, the MIC estimates for the composite scores were 0.51 or below, with the upper limits of the 95% confidence intervals at 0.63 or below. Therefore, the minimally important individual-level worsening in a PRO-CTCAE composite score would also be a one-point increase.

The magnitudes of the MICs in PRO-CTCAE observed scores and composite scores were somewhat smaller in the PRO-TECT data compared to the validation data, even though the proportion of patients who worsened on anchor measures was very similar between the two datasets, as shown in Figs. 1 and 2. If there were more ceiling effects in the PRO-TECT data, with a higher proportion of respondents reporting extreme symptoms at the top of the scale, there would be little to no room for them to report further decline. However, there was little evidence of ceiling effects in either dataset: in the PRO-TECT data, 0.2% to 7.1% of patients selected the response option indicating the worst symptoms, and in the validation data, the range was 0.1% to 7.0%.

A possible explanation for the variability in MICs could be differences in tumor site, treatment type, performance status, or other sociodemographic characteristics. For instance, the patients in the PRO-TECT data were slightly older on average (61.7 compared to 58.0), had a higher proportion of female patients (62.8% compared to 56.3%), a higher proportion of white patients (80.0% compared to 72.1%), a lower proportion of college graduates (24.4% compared to 44.4%). Clinically, all patients in the PRO-TECT trial had metastatic cancer, and there were higher proportions of patients with lung or GI cancer compared to those in the validation study. Treatment characteristics were not directly comparable, because the PRO-TECT collected information on lines of systemic cancer treatment, including intravenous and oral delivery methods, whereas the validation data collected information on RT, surgery, and chemotherapy.

Alternatively, the smaller MICs in the PRO-TECT trial could be due to the study design, which incorporated alerts to patients whenever they reported a concerning symptom. The magnitude of change scores on the anchor measures among the decliners were very similar between the two datasets. For example, the mean score change among decliners on the EORTC QLQ-C30 constipation scale was 38.3 (SD of the change score = 15.6, n = 67) in the PRO-TECT data, which was very similar to 40.6 (SD of the change score = 16.0, n = 143) in the validation data (*t* = 0.99, two-tailed *p* = 0.33). Among these patients who meaningfully worsened on the EORTC constipation anchor, those in the PRO-TECT trial had a much smaller average change (0.22) in the PRO-CTCAE constipation (S) item compared to the validation data (0.91). Accordingly, the MIC estimate for constipation (S) item was small (0.10) in the PRO-TECT data compared to the validation data (0.49). Supplementary Figs. 1 and 2 illustrate the PRO-CTCAE score distributions at baseline and follow-up for both data sources. Notably, there was a higher prevalence of zeros and ones at follow-up in the PRO-TECT trial.

Patients in the PRO-TECT trial stayed on study for a median duration of 11.3 months, reporting symptoms weekly based on PRO-CTCAE for remote symptom monitoring, which generated alerts for severe or worsening symptoms. Interestingly, only 10% of these alerts were considered urgent by the respondents, warranting immediate contact with the cancer care team rather than waiting for the next appointment [21]. This might have influenced how patients reported their symptoms, potentially moderating their responses based on their perception of symptom severity and the need for alerts. Consequently, data collected for different purposes, such as validation versus remote symptom monitoring, could impact MIC estimates. This underscores the importance of employing multiple studies, methods, and populations to enhance our understanding and confidence in MIC estimates [6].

Because the ten EORTC QLQ-C30 scales used as anchors in this study are comprised of one to four items, with each item taking values from 1 to 4, the measurement scale is ordinal, with change increments (single response category movement on an item) ranging from 8.3 to 33.3 [22]. Notably, to be categorized as meaningfully changed, emotional functioning requires a score change of 16.6, corresponding to two-increment change, while other scales require one change increment, ranging from 11.1 to 33.3. Implementing the 10-point threshold, the smallest change categorized as meaningful was seen in the fatigue scale (11.1), followed by emotional functioning (16.6), cognitive functioning (16.7), nausea/vomiting (16.7), and pain (16.7). This could explain the smaller MICs observed in fatigue (0.19 and 0.24), anxiety (0.30 to 0.37), concentration (0.25 and 0.26), and pain (0.30 to 0.34). For PRO-CTCAE items matched with single-item anchor measures with a larger increment (33.3), MIC estimates in the validation data ranged from 0.35 to 0.53, averaging 0.45. Therefore, anchor measures that allow for more granular increments of change might yield smaller MIC estimates, making the current MIC estimates conservative.

An important caveat of the current study lies in its focus on a subset of PRO-CTCAE items that are correlated with EORTC QLQ-C30 scales. This investigation employed EORTC QLQ-C30 scales and interpreted a ≥ 10-point change as reflecting meaningful worsening. Future studies could explore alternative anchors, such as patients’ global ratings of change or clinical anchors measured at baseline and later follow-up. Future studies should employ other methods and larger samples in different trial contexts to confirm and extend our observations. Lastly, the FDA [23, 24] has recommended applying both qualitative and quantitative research methods to study and triangulate data on what is important to patients. This approach opens the door for further research into eliciting patient definitions or evaluations of meaningful changes on PRO-CTCAE items through interviews, focus groups, or surveys [25–28], ensuring a comprehensive understanding by integrating multiple data sources.

## Conclusion

This study derived meaningful change thresholds of worsening for select PRO-CTCAE items and composite scores. In general, the meaningful individual-level change threshold for worsening was found to be one point. Our examination of meaningful change was based on statistical criteria, and further research is required to validate or bolster confidence in these thresholds. Subsequent studies should also explore clinically meaningful change that indicates a change in the therapeutic treatment or in the prognosis of the disease, considering its real-world impact on patients’ well-being and health status.

## Supplementary Information

Below is the link to the electronic supplementary material.

Supplementary file1 (DOCX 1797 KB)
