# Supplementary material for: Identification of meaningful individual-level change thresholds for worsening on the patient-reported outcomes version of the common terminology criteria for adverse events (PRO-CTCAE®)
Source: Qual Life Res. 2024 Nov 6;34(2):495–507. doi: 10.1007/s11136-024-03819-5 (PMC11865169; doi:10.1007/s11136-024-03819-5)
Supplement: Supplementary file 1 — Supplementary file1 (DOCX 1797 KB) [file 11136_2024_3819_MOESM1_ESM.docx]

Supplementary table 1. PRO-TECT data: Spearman correlations between the PRO-CTCAE change scores and EORTC QLQ-C30 change scores at 3-month follow-up


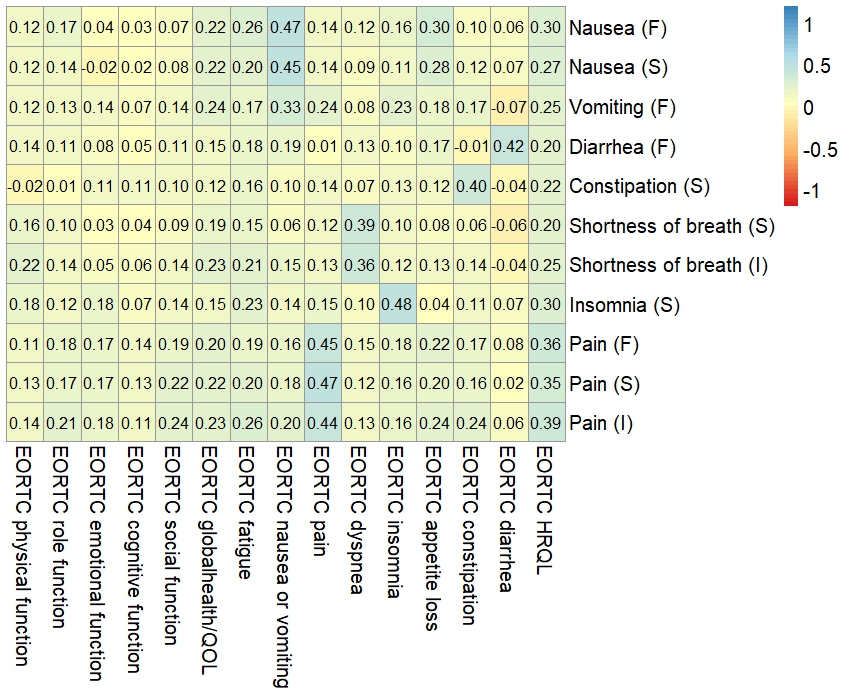


Note. All EORTC scales were coded such that higher scores indicate worse symptoms, functioning, and quality of life. The change scores were from baseline to 3-month follow-up. For PRO-CTCAE, “F” stands for frequency. “S” stands for severity, and “I” stands for interference. Based on these correlations, the minimally important changes (MICs) in the following items were investigated in the PRO-TECT data: Pain (F, S, I), nausea (F, S), vomiting (F), constipation (S), diarrhea (F), shortness of breath (S, I), and insomnia (S).

Supplementary Table 2. Validation data: Correlations between the PRO-CTCAE change scores and EORTC change scores (PRO-CTCAE items for which at least 85% of the patients have change scores were included in the correlation analysis)


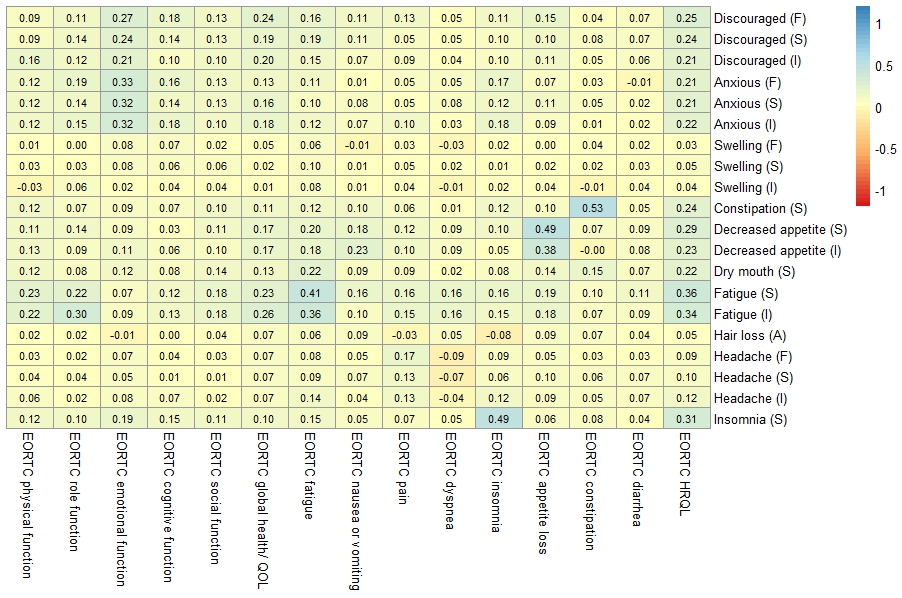


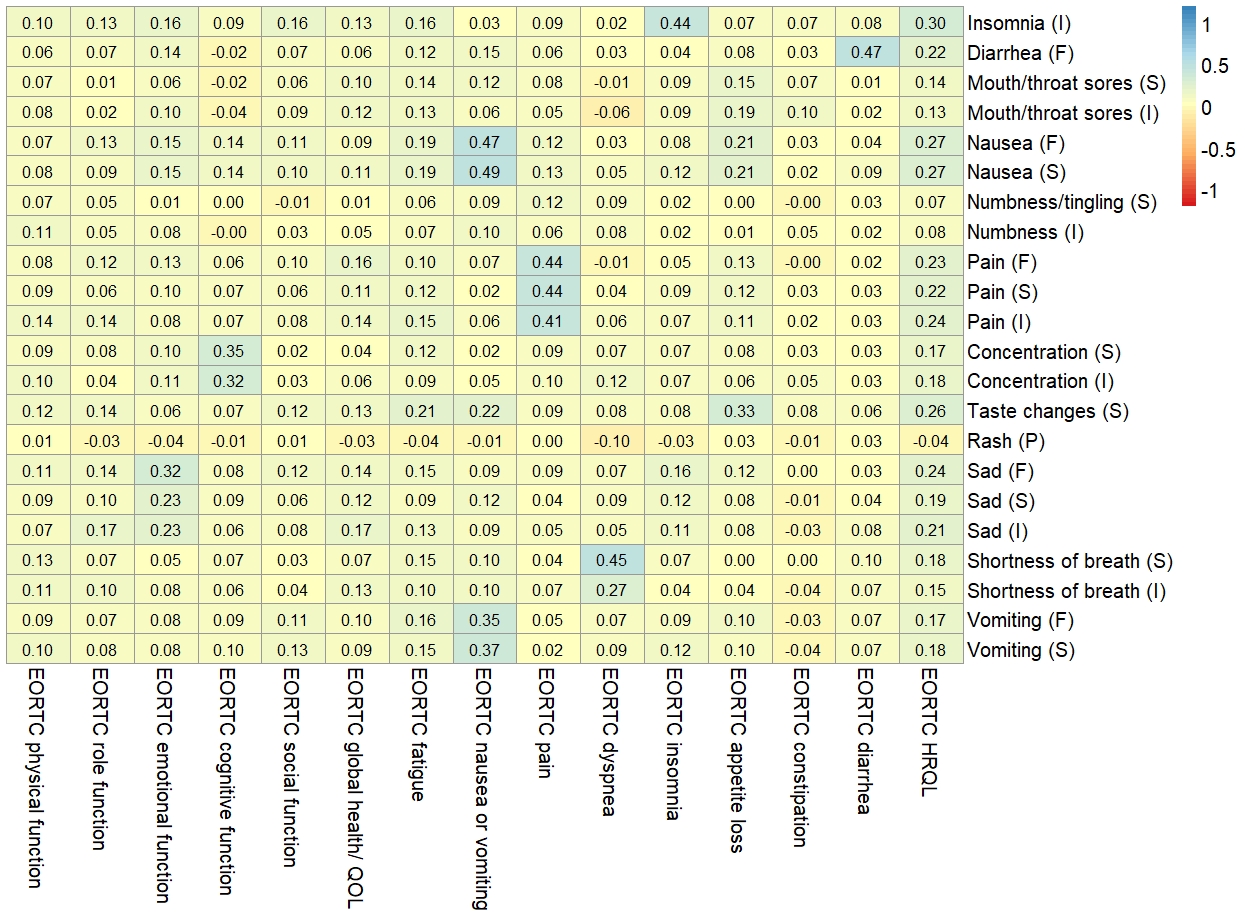


Note. All EORTC scales were coded such that higher scores indicate worse symptoms, functioning, and quality of life. PRO-CTCAE items in the validation data had either > 50% missing response rates or <15% missing responses, due to the original study design, and we analyzed items that had <15% missing data in the current study. Based on the response rates and correlations, the minimally important changes (MICs) in the following items were investigated in the validation data: Anxious (F, S, I), constipation (S), decreased appetite (S, I), fatigue (S, I), insomnia (S, I), diarrhea (F), nausea (F, S), pain (F, S, I), concentration (S, I), taste changes (S), sad (F), shortness of breath (S), and vomiting (F, S).

Supplementary Table 3. Average item-level PRO-CTCAE change scores for EORTC decliners

| Anchor: EORTC QLQ-C30 scale | PRO-CTCAE item | PRO-TECT data | | Validation data | |
| --- | --- | --- | --- | --- | --- |
|  |  | Average change | N | Average change | N |
| Emotional functioning | Anxious (F) |  |  | 0.49 | 118 |
|  | Anxious (S) |  |  | 0.40 | 118 |
|  | Anxious (I) |  |  | 0.39 | 118 |
|  | Sad (F) |  |  | 0.32 | 120 |
| Constipation | Constipation (S) | 0.22 | 67 | 0.91 | 137 |
| Appetite loss | Decreased appetite (S) |  |  | 0.91 | 139 |
|  | Decreased appetite (I) |  |  | 0.62 | 139 |
|  | Taste changes (S) |  |  | 0.52 | 139 |
| Fatigue | Fatigue (S) |  |  | 0.52 | 265 |
|  | Fatigue (I) |  |  | 0.47 | 264 |
| Insomnia | Insomnia (S) | 0.34 | 62 | 0.73 | 140 |
|  | Insomnia (I) |  |  | 0.59 | 139 |
| Diarrhea | Diarrhea (F) | 0.51 | 68 | 0.96 | 92 |
| Nausea or vomiting | Nausea (F) | 0.44 | 61 | 0.82 | 114 |
|  | Nausea (S) | 0.36 | 61 | 0.79 | 114 |
|  | Vomiting (F) | 0.33 | 61 | 0.38 | 113 |
|  | Vomiting (S) |  |  | 0.39 | 113 |
| Pain | Pain (F) | 0.34 | 119 | 0.94 | 210 |
|  | Pain (S) | 0.35 | 119 | 0.62 | 210 |
|  | Pain (I) | 0.38 | 119 | 0.57 | 209 |
| Cognitive functioning | Concentration (S) |  |  | 0.49 | 175 |
|  | Concentration (I) |  |  | 0.45 | 175 |
| Dyspnea | Shortness of breath (S) | 0.53 | 87 | 0.57 | 101 |
|  | Shortness of breath (I) | 0.49 | 87 | * | |

Note. Using the anchors of EORTC QLQ-C30 scales, those who worsened by 10 points or more were categorized as decliners. The average changes of the selected PRO-CTCAE items in the PRO-TECT data for decliners ranged from 0.22 to 0.53 with mean of 0.39 (median: 0.36). Those in the validation data ranged from 0.32 to 0.96 with mean of 0.61 (median: 0.57). The blanks in the table show the PRO-CTCAE items that we did not administer in the PRO-TECT trial. The * shows the item that had a low correlation (≤ .30) with the anchor.

Supplementary Table 4. Average changes in in PRO-CTCAE composite scores for EORTC decliners

| Anchor: EORTC QLQ-C30 scale | PRO-CTCAE composite score | PRO-TECT data | | Validation data | |
| --- | --- | --- | --- | --- | --- |
|  |  | Average change | N | Average change | N |
| Emotional functioning | Anxious |  |  | 0.43 | 138 |
| Constipation | Constipation | 0.19 | 67 | 0.85 | 143 |
| Appetite loss | Decreased appetite |  |  | 0.69 | 154 |
|  | Taste changes |  |  | 0.50 | 154 |
| Fatigue | Fatigue |  |  | 0.41 | 286 |
| Insomnia | Insomnia |  |  | 0.62 | 154 |
| Diarrhea | Diarrhea | 0.35 | 68 | 0.63 | 104 |
| Nausea or vomiting | Nausea | 0.38 | 61 | 0.72 | 133 |
|  | Vomiting |  |  | 0.36 | 133 |
| Pain | Pain | 0.33 | 119 | 0.59 | 231 |
| Cognitive functioning | Concentration |  |  | 0.42 | 194 |
| Dyspnea | Shortness of breath | 0.47 | 87 | 0.50 | 119 |

Note. Using the anchors of EORTC QLQ-C30 scales, those who worsened by 10 points or more were categorized as decliners. The average changes of the selected PRO-CTCAE composite scores for decliners in the PRO-TECT data ranged from 0.19 to 0.47 with mean of 0.34 (median: 0.35). Those in the validation data ranged from 0.36 to 0.85 with mean of 0.56 (median: 0.55). Those The blanks in the table show the composites in the PRO-TECT data, for which we did not have the complete set of items in the dataset.

Supplementary Figure 1. **Baseline** PRO-CTCAE score distribution

(a) PRO-TECT data

(b) Validation data (only showing common items with PRO-TECT)

Supplementary Figure 2. **Follow-up** PRO-CTCAE score distribution

(a) PRO-TECT data

(b) Validation data (only showing common items with PRO-TECT)
